# Supplementary material for: N‐acetylaspartate release by glutaminolytic ovarian cancer cells sustains protumoral macrophages
Source: EMBO Rep. 2021 Jul 14;22(9):e51981. doi: 10.15252/embr.202051981 (PMC8419692; doi:10.15252/embr.202051981)
Supplement: Supplementary file 2 — Expanded View Figures PDF [file EMBR-22-e51981-s002.pdf]

Expanded View Figures

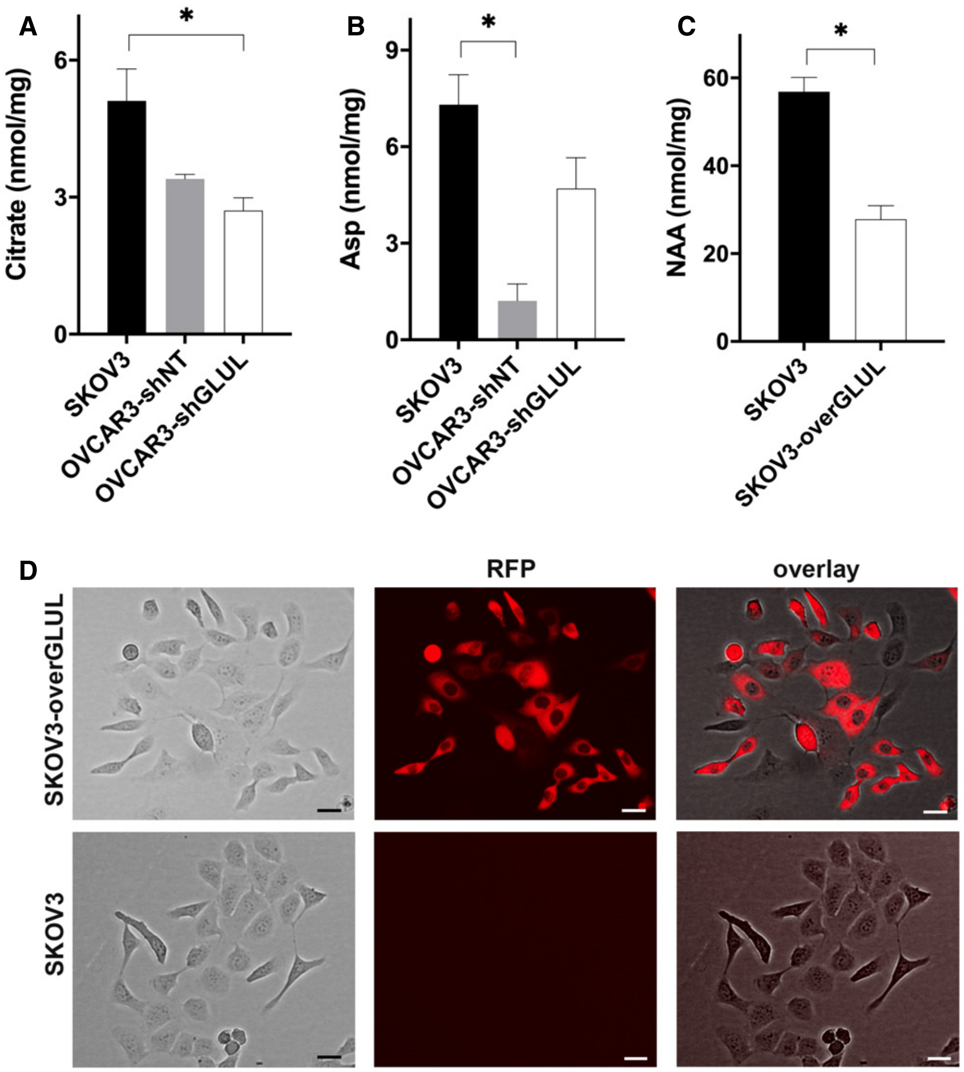

Figure EV1. SKOV3 metabolic reprogramming mimics that of OVCAR3-sh*GLUL* cells.

A, B Intracellular levels of (A) citrate, (B) aspartate (Asp) in SKOV3 cells compared with OVCAR3-shNT and OVCAR3-sh*GLUL* cells (n = 3 biological replicates).

C NAA levels in SKOV3 cells compared with SKOV3-over-*GLUL* cells (n = 6 biological replicates). Metabolite levels are all measured with LC-MS/MS analysis after 84-h incubation.

D Transduction of SKOV3 cells verified by the RFP fluorescent signal. The images were collected by CELENA® S Digital Imaging System at 10× magnification (n = 3 biological replicates). The scale bars indicate 25 μm.

Data information: Data are displayed as mean ± SEM. Statistical significance was calculated by one-way ANOVA analyses with Tukey correction (A, B), unpaired t-test (C) and defined as $*P < 0.05$.

© 2021 The Authors

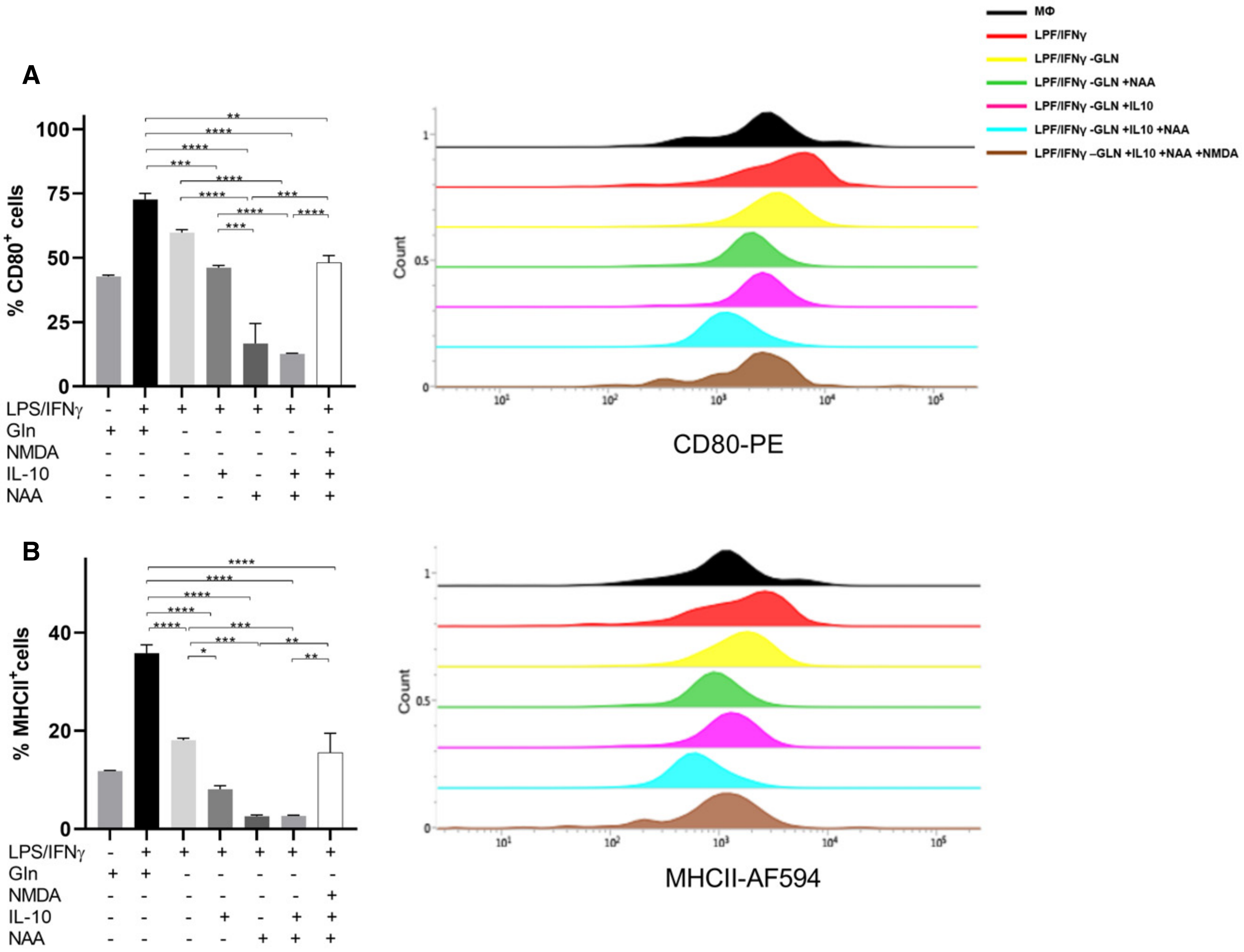

Figure EV2. Effect of NAA on macrophage polarization by FACS.

A, B Flow cytometric quantification of the percentage of CD80$^+$(A) and MHCII$^+$ (B) cells after specific treatments. Mφ macrophages were used as a control. Representative overlaid flow cytometry histograms normalized to cell count (*n* = 3 biological replicates) are shown.

Data information: Data are displayed as mean ± SEM. Statistical significance was calculated by one-way ANOVA analyses with Tukey correction and defined as **P* < 0.05, ***P* < 0.01, ****P* < 0.001, *****P* < 0.0001.

Figure EV3. Effect of NAA on macrophages in different conditions.

A qRT–PCR quantification of *ASPA* mRNA levels in LPS/IFNγ macrophages treated with NAA (10 μM) and/or IL-10 and/or NMDA and/or with si*ASPA* (*n* = 3 biological replicates).

B–E qRT–PCR quantification of (B) *CD206*, (C) *CD163*, (D) *CD80*, and (E) *GLUL* mRNA levels in resting (Mφ) macrophages and LPS/IFNγ macrophages treated with NAA (10 μM) and/or IL-10 and/or NMDA (*n* = 3 biological replicates) in the presence of Gln.

F–I Comparison between qRT–PCR quantifications of (F) *CD206*, (G) *CD163*, (H) *CD80*, and (I) *GLUL* mRNA levels in LPS/IFNγ macrophages treated with NAA (10 μM) and/or IL-10 and/or NMDA (*n* = 3 biological replicates) in the absence of Gln, with qRT–PCR quantifications of the same markers in LPS/IFNγ macrophages treated with NAA (10 μM) and/or IL-10 and/or NMDA (*n* = 3 biological replicates) in the presence of Gln (*n* = 3 biological replicates).

J–L qRT–PCR quantification of (J) *CD206*, (K) *CD163*, (L) *GLUL* mRNA levels in IL-10 macrophages treated with NAA (10 μM) and/or NMDA (*n* = 3 biological replicates).

Data information: Data are displayed as mean ± SEM. For (A-E, J-L) panels, statistical significance was calculated by one-way ANOVA analyses with Tukey correction, for (F-I) panels by unpaired *t*-test and defined as **P* < 0.05, ***P* < 0.01, ****P* < 0.001, *****P* < 0.0001.

 © 2021 The Authors

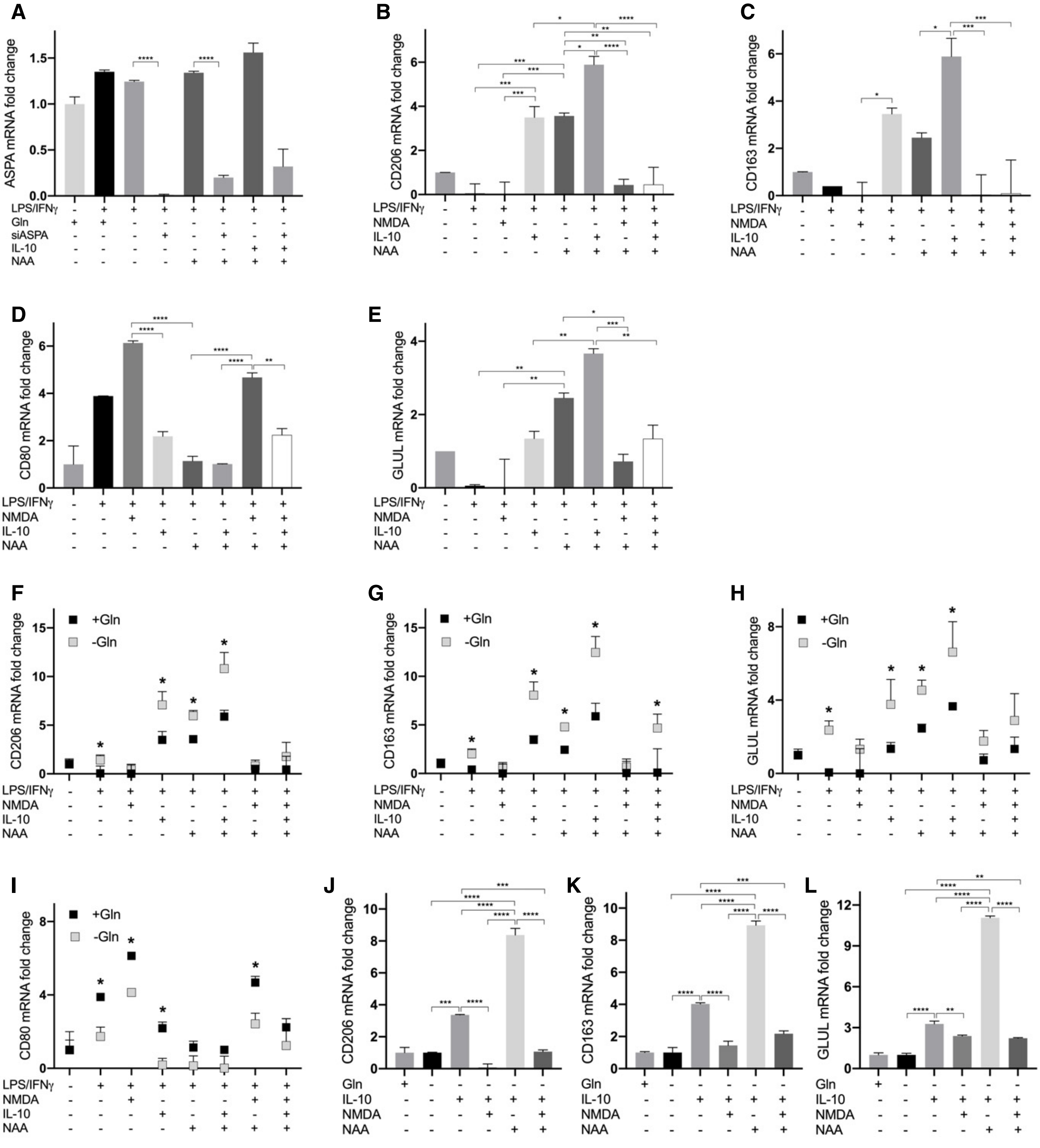
A
ASPA mRNA fold change
B
CD206 mRNA fold change
C
CD163 mRNA fold change
D
CD80 mRNA fold change
E
GLUL mRNA fold change
F
CD206 mRNA fold change
G
CD163 mRNA fold change
H
GLUL mRNA fold change
I
CD80 mRNA fold change
J
CD206 mRNA fold change
K
CD163 mRNA fold change
L
GLUL mRNA fold change
+Gln
-Gln
LPS/IFNγ
Gln
siASPA
NMDA
IL-10
NAA

Figure EV3.

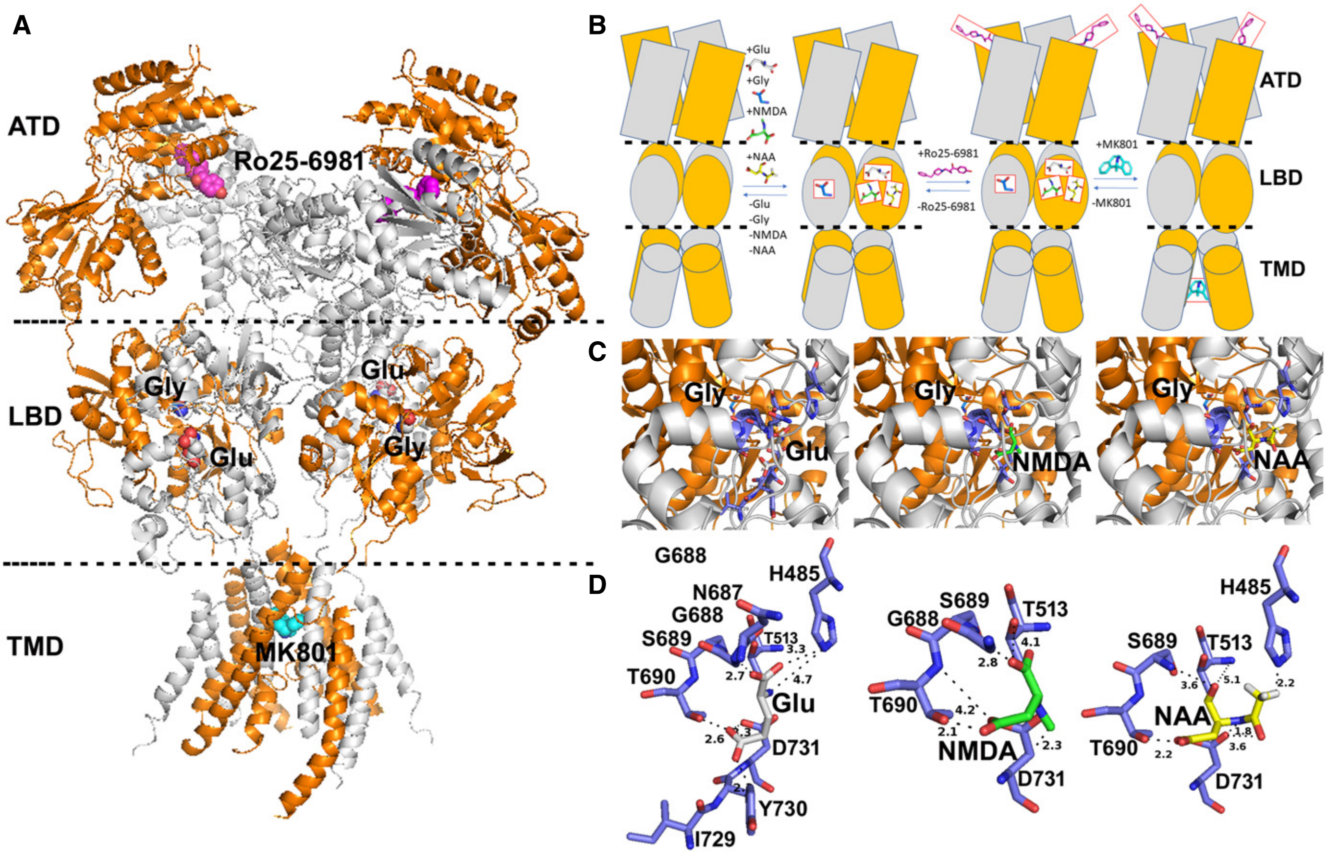

Figure EV4. Lateral view of the human GluN1/GluN2 NMDAR in the glutamate/glycine bound state and interactions between NMDAR and Glu, NMDA, and NAA ligands.

A Lateral view of the human cryo-EM solved NMDAR structure (6irg.pdb), consisting of 2 GluN1 subunits (white cartoon) and 2 GluN2A (orange cartoon) in complex with Glu, Gly, Ro25-6981, and MK-801 ligands, reported in white, magenta, and cyan sphere representation, respectively.

B Scheme representation of the human NMDAR interacting with agonists and/or inhibitors.

C, D Modeling and zoomed views of the GluN2D ligand-binding core showing residues involved in direct interactions with Glu (white sticks), NMDA (green sticks), and NAA (yellow sticks).

Figure EV5. Ascitic and cancer tissue arrays *ASPA*, GS, GLS1, Ki67, and CD163 evaluations.

A qRT–PCR quantification of *ASPA* mRNA levels in TAMs isolated from ovarian cancer patients (n = 53, 5 of stage I; 7 of stage II; 28 of stage III; 13 of stage IV) versus resting (Mφ) macrophages.

B Gln levels in ascitic fluid isolated from ovarian cancer patients (n = 38, 4 of stage I; 7 of stage II; 20 of stage III; 7 of stage IV) determined with LC-MS/MS analysis.

C, D The ovary cancer tissue arrays (US BioMax) containing 70 (OV701) and 99 (OV991) cases (n = 58 of stage I, 24 of stage II, 24 of stage III, and 2 of stage IV) were stained with anti-GS, *GLS1*, Ki67, and CD163. The representative merged tissue images ("merge" panels, also shown in Fig 5G) are shown here together with those in splitted channels. The images were collected at 20× magnification using Nikon Eclipse 80i-ViCO. The scale bars indicate 100 μm.

Data information: Data are displayed as mean ± SEM. For all panels, statistical significance was calculated by one-way ANOVA analyses with Dunn's correction.

 © 2021 The Authors

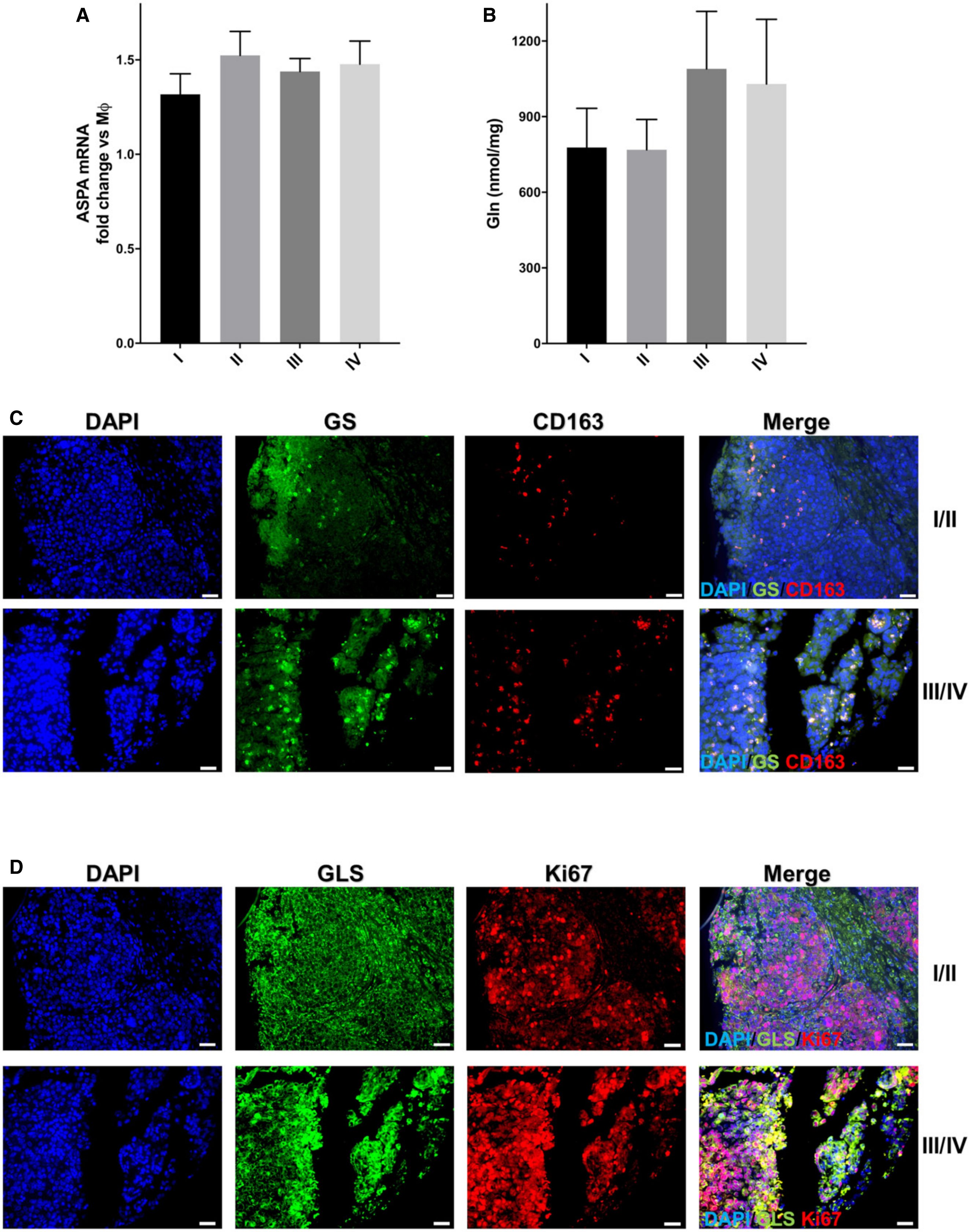
A
ASPA mRNA fold change vs Mϕ
0.0
0.5
1.0
1.5
I
II
III
IV
B
Gln (nmol/mg)
0
300
600
900
1200
I
II
III
IV
C
DAPI
GS
CD163
Merge
DAPI/GS/CD163
I/II
III/IV
D
DAPI
GLS
Ki67
Merge
DAPI/GLS/Ki67
I/II
III/IV

Figure EV5.

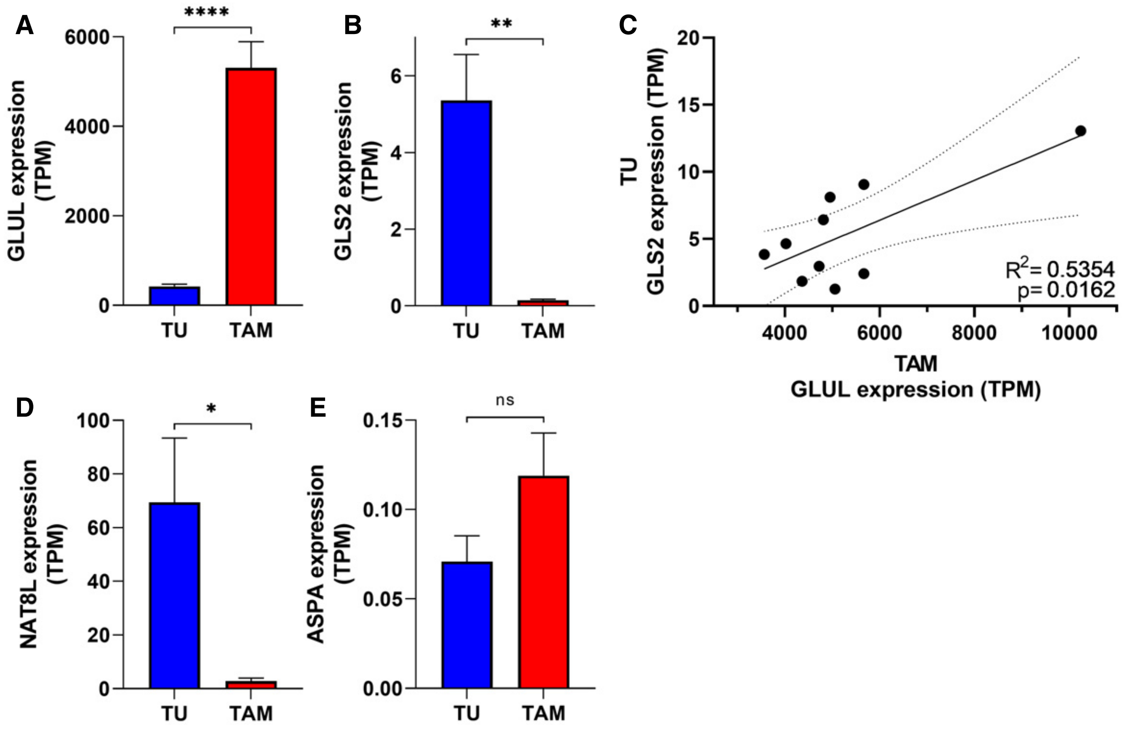

Figure EV6. RNA-seq data for ascitic tumor cells (TU) and TAM-matched samples from HGSOC patients (stage III).

A, B Expression of (A) *GLUL* and (B) *GLS2* in ascitic tumor cells (TU) compared with ascite-associated macrophages (TAM) (n = 10 patients).

C Scatter plot shows the correlation of *GLS2* expression in tumor cells with *GLUL* expression in TAM. Dashed lines indicate 95% confidence intervals. R: Pearson correlation coefficients; R^2: means "the goodness of fit" (n = 10 patients).

D, E Expression of (D) *NAT8L* and (E) *ASPA* in ascitic tumor cells (TU) compared with ascite-associated macrophages (TAM) (n = 10 patients).

Data information: Transcripts per million (TPM) values were used as measure of mRNA expression. Data are displayed as mean ± SEM. Statistical significance was calculated by paired t-test (A, B, D, E), Pearson correlation coefficients (C) and defined as $*P < 0.05$, $**P < 0.01$, $****P < 0.0001$, ns, not significant.

© 2021 The Authors
